# Glioblastoma biomarkers in urinary extracellular vesicles reveal the potential for a ‘liquid gold’ biopsy

**DOI:** 10.1038/s41416-023-02548-9

**Published:** 2024-01-11

**Authors:** Susannah M. Hallal, Ágota Tűzesi, Liam A. Sida, Elissa Xian, Daniel Madani, Krishna Muralidharan, Brindha Shivalingam, Michael E. Buckland, Laveniya Satgunaseelan, Kimberley L. Alexander

**Affiliations:** 1 Brain Cancer Research, Neurosurgery Department, Chris O’Brien Lifehouse, Camperdown, NSW Australia; 2 Department of Neuropathology, Royal Prince Alfred Hospital, Camperdown, NSW Australia; 3 School of Medical Sciences, Faculty of Medicine and Health Sciences, The University of Sydney, Camperdown, NSW Australia; 4 Neurosurgery Department, Royal Prince Alfred Hospital, Camperdown, NSW Australia; 5 Sydney Medical School, Faculty of Medicine and Health Sciences, The University of Sydney, Sydney, NSW Australia

**Keywords:** Tumour biomarkers, Proteomics, Diagnostic markers, CNS cancer

## Abstract

**Background:**

Biomarkers that reflect glioblastoma tumour activity and treatment response are urgently needed to help guide clinical management, particularly for recurrent disease. As the urinary system is a major clearance route of circulating extracellular vesicles (EVs; 30–1000 nm nanoparticles) we explored whether sampling urinary-EVs could serve as a simple and non-invasive liquid biopsy approach for measuring glioblastoma-associated biomarkers.

**Methods:**

Fifty urine specimens (15–60 ml) were collected from 24 catheterised glioblastoma patients immediately prior to primary (*n* = 17) and recurrence (*n* = 7) surgeries, following gross total resection (*n* = 9), and from age/gender-matched healthy participants (*n* = 14). EVs isolated by differential ultracentrifugation were characterised and extracted proteomes were analysed by high-resolution data-independent acquisition liquid chromatography tandem mass spectrometry (DIA-LC-MS/MS).

**Results:**

Overall, 6857 proteins were confidently identified in urinary-EVs (*q*-value ≤ 0.01), including 94 EV marker proteins. Glioblastoma-specific proteomic signatures were determined, and putative urinary-EV biomarkers corresponding to tumour burden and recurrence were identified (FC ≥ | 2 | , adjust *p*-val≤0.05, AUC > 0.9).

**Conclusion:**

In-depth DIA-LC-MS/MS characterisation of urinary-EVs substantiates urine as a viable source of glioblastoma biomarkers. The promising ‘liquid gold’ biomarker panels described here warrant further investigation.

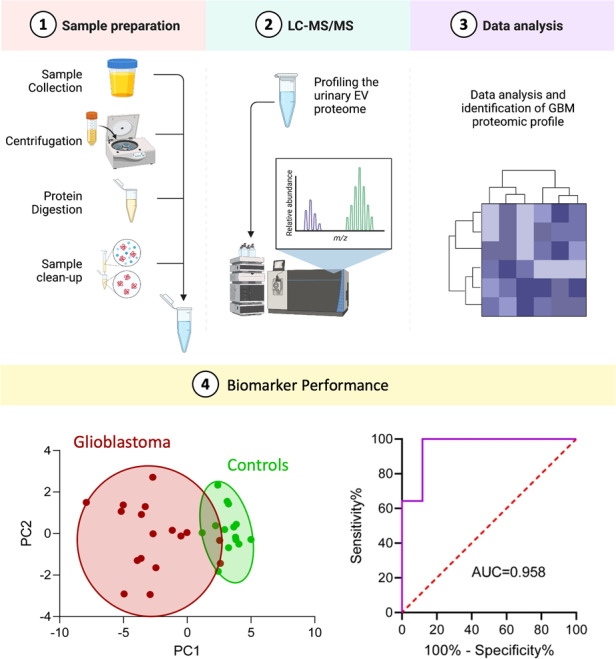

## Background

Improving outcomes for people diagnosed with glioblastoma (GBM), IDH-wildtype (IDHwt), the most common and lethal primary brain tumour in adults, requires the development of sensitive methods that efficiently and sensitively monitor tumour activity and treatment response. Following standard treatment, almost all GBMs recur, and when they do, they are often rapidly fatal. Monitoring GBM patients for tumour recurrence and assessing treatment efficacy is frequently challenged and confounded by radiological mimics of tumour progression, termed ‘pseudoprogression’. The lack of reliable tumour surveillance methods often impacts timely adjustments to treatment schedules and can lead to the premature cessation of treatment and even unnecessary neurosurgeries, that only worsen patient outcomes.

Liquid biopsies that measure tumour-derived factors in body fluids offer new avenues for monitoring tumour evolution in real-time. As such, the development of novel liquid biopsy strategies that measure sensitive and specific biomarkers corresponding to tumour activity and treatment response(s) are a research priority in neuro-oncology. We, and others, have explored extracellular vesicles (EVs) as reservoirs of GBM biomarkers in patient body fluids [1–5] and a robust GBM signal has been detected in EVs from neurosurgical fluids [3, 4], cerebrospinal fluid (CSF) [6, 7] and peripheral blood [1, 5]. GBM cells release an enormous number of EVs in vivo with a single GBM cell secreting approximately 10,000 EVs over a 48 h period [8]. EVs released by GBM tumours can cross the blood-brain-barrier into the circulation where they carry molecules that reflect the active state of parent cells [9, 10]. As the urinary system is a major clearance route of circulating EVs, we hypothesised that GBM biomarkers are also assessable in urinary-EVs (uEVs).

Reports of uEV-associated biomarker studies predominantly relate to diseases of the bladder and kidney [11–13], however, uEV cargoed miRNAs were shown to discriminate breast cancer patients from healthy controls [14]. uEVs have also shown promise as biomarkers for neurological diseases with a striking enrichment of proteins linked to Parkinson’s, Alzheimer’s, and Huntington’s disease [7, 15–17]. A uEV protein signature was strongly associated with a Parkinson’s diagnosis as well as the severity of cognitive impairment [16], while toxic beta and tau proteins integral to Alzheimer’s disease (AD) pathology were significantly higher in uEVs from AD patients relative to healthy controls [17]. AD patients have higher numbers of EVs in their urine and studies suggest that differences in uEV quantity and cargoed molecules may provide a basis for early diagnosis of AD [17]. Only one study has examined GBM uEVs in the literature to date. Here, a nanowire assay system measured CD31:CD63 surface expression differences between GBM patients and healthy individuals [18]. While no comprehensive GBM uEV biomarker discoveries have been reported, GBM biomarkers have been resolved in unfractionated urine. An early study investigated the utility of urinary matrix metalloproteinases (MMPs) as diagnostic biomarkers of GBM and showed that significantly elevated urine MMP levels correlate with the presence of GBM, with MMP levels decreasing during treatment [19]. More recently, Wu et al., employed a proteomics strategy to identify biomarkers in urine collected from GBM patients at the time of tumour diagnosis and after surgical resection of the tumour, and identified 27 soluble urinary proteins functionally associated with autophagy and angiogenesis, both important in tumour development [20].

Although EV-associated proteins are highly suitable biomarkers, comprehensive proteomic characterisation of EVs derived from body fluids is challenging. For instance, urine contains the highly-abundant protein, uromodulin [21, 22], that commonly co-isolates with EVs and can mask the detection of less-abundant, potential EV-biomarker proteins in shotgun liquid chromatography coupled tandem mass spectrometry (LC-MS/MS) analyses [23, 24]. New strategies to analyse complex biological samples preferentially use highly-specific data independent acquisition (DIA) MS [25], a label-free method that allows the identification and quantification of all peptides within a sample. Here, all ionised compounds within a sample that fall within a specified mass-range (approximately 25 Da) are fragmented in an unbiased fashion so that all ions undergo MS/MS [26–28]. Maximal proteome coverage can be obtained by aligning DIA-MS data to a high-quality, comprehensive spectral library that contains MS coordinates for target peptides, consisting of (i) the peptide precursor ion *m/z*, (ii) the *m/z* of the fragment ions and their intensities and (iii) the chromatographic retention time of the peptide [29]. This information allows DIA analysed peptides to be identified and quantified if they are present in the library. High-resolution extracted ion chromatograms (XICs) are drawn for every peptide in the sample, enabling sensitive and accurate quantitation, even for low abundant peptides [29].

In this study, we assess the feasibility of utilising small uEVs (<300 nm) as a simple and non-invasive liquid biopsy approach for measuring GBM biomarkers—a ‘*liquid gold biopsy’*. Using a DIA-MS approach in conjunction with a highly specific GBM-EV spectral library, we present the first in-depth proteomic composition of uEVs from GBM patients and identify putative biomarker panels corresponding to a GBM diagnosis, tumour load and recurrent progression. Furthermore, we explore whether previously defined GBM EV biomarkers from other body compartments, i.e., the central nervous system and blood circulation, are also reflected in patient urine.

## Materials and methods

### Cohort information and urine collection

Urine specimens were collected from catheterised GBM patients and stored at −80 °C by the Sydney Brain Tumour Bank (SLHD HREC X19-0010). This biomarker discovery study was performed under approved human ethics protocol USYD HREC 2019/705. Urine (20–100 ml) was collected from participants formally diagnosed with Glioblastoma, IDH-wildtype, CNS WHO grade 4 (2021) [30] at three distinct clinical timepoints, i.e., before (Pre-OP; *n* = 17) and after surgical removal of primary tumours (Post-OP; *n* = 9 matched samples), and prior to the surgical removal of a GBM recurrence (REC; *n* = 7). All Pre-OP urine specimens were collected immediately prior to surgery and paired Post-OP samples collected the day following a gross total resection (average 17.8 h post-surgery). Nearly all GBM patients (23/24) had normal renal function. Pre- and post-operative urea-creatine ratios were similar in all patients indicating stable perioperative hydration coinciding with the urine sampling timepoints. GBM urine samples were compared to urine from age- and gender-matched healthy controls, collected as mid-stream, first morning pass urine specimens (HC; *n* = 14). A summary of experimental cohorts analysed here is provided in Table 1; refer to Supplementary Table 1 for additional demographic and clinical detail.

**Table 1:** Summary of Experimental Cohorts.

Experimental cohorts	Sample ‘*n*’	Mean age (range)	Gender
Pre-OP, primary GBM IDHwt	17	61.9 (42–92)	10 M/7 F
Post-OP, primary GBM IDHwt (*matched*)	9	56.7 (42–76)	7 M/2 F
REC, recurrent GBM IDHwt	7	58.1 (27–77)	3 M/4 F
Healthy controls, non-cancer volunteers	14	69.4 (63–75)	7 M/7 F

### Isolation and characterisation of urinary-EVs

To isolate EVs, thawed urine samples (20–100 mL) were subjected to a differential ultracentrifugation protocol (Supplementary Fig. 1). Briefly, an initial 3,000 x *g* centrifugation step was used to pellet cell debris and larger particles and the supernatant was kept for further processing. The pellet was treated with 10 mM TCEP-HCl/100 mM Tris-HCl/50 mM sucrose (15 min at RT) and then diluted with 1.2 ml 4 mM TCEP-HCl/10 mM Tris/HCl followed by 17,000 x *g* centrifugation (30 min, 4 °C) to pellet large EVs (l-EVs; *not studied here*). The TCEP-treated supernatant was then combined with the initial supernatant (above) and subject to 100,000 x *g* ultracentrifugation (2 h, 4 °C) to pellet small EVs (fixed angle rotor F-37L-8 8x100ml, THERMO WX 100). Isolated small EV populations were characterised according to the latest guidelines of the International Society for Extracellular Vesicles on the Minimal Information Required for Studies of Extracellular Vesicles (MISEV2018) [31]. EV size distributions and concentrations were measured by nanoparticle tracking analysis software (NTA, version 3.0) using the NanoSight LM10-HS (NanoSight Ltd., Amesbury, UK), configured with a 532-nm laser and a digital camera (CMOS Trigger Camera). EVs were diluted in filtered PBS (viscosity 1.09 cP) to ensure that 20–100 particles were detected in the field of view in the standard CCD camera of the microscope. The NTA video recordings (60 s) were captured in triplicate at 25 frames/s with default minimal expected particle size, minimum track length, and blur setting, a camera level of 11 and detection threshold of 5. The temperature of the laser unit was controlled at 25 °C. NTA software measured the size distribution (ranging from 10 to 1000 nm) and concentration (particles/ml) of nanoparticles by simultaneously tracking Brownian motion and light scatter of individual laser-illuminated particles and calculated their diameter using statistical methods [32]. EV samples were imaged by cryogenic-transmission electron microscopy (cryo-TEM), as previously described [33] (Sydney Microscopy and Microanalysis, University of Sydney). Briefly, the EV samples were applied to copper 300-mesh lacey carbon grids and plunged frozen into ethane using a Vitrobot IV (ThermoFisher). The grids were imaged using SerialEM (Mastronarde) on a ThermoFisher Glacios (operated at 200 kV) equipped with a Falcon III camera (ThermoFisher) at 45,000x magnification. Image scale bars were determined in ImageJ 1.53 K software. Lastly, LC-MS/MS data was used to identify canonical EV markers and the presence of the top 100 EV-marker proteins as curated in Vesiclepedia, a compendium of EV-associated molecules.

### EV proteome preparation for LC-MS/MS

uEV proteomes were extracted and prepared for LC-MS/MS analysis using established methods [3]. Briefly, the EV pellets were resuspended in 0.2% (*w/v*) Rapigest SF^TM^ (Waters, Milford, MA, USA) in 0.05 mol/L triethylammonium bicarbonate (TEAB), incubated at 95 °C for 5 min and sonicated twice with a step-tip probe at 30% intensity for 20 s to aid EV lysis and protein resuspension. The protein content of the uEV pellets were estimated with a Qubit® Protein Assay Kit (Invitrogen, Carlsbad, CA, USA) and 25–50 μg EV protein aliquots were digested by sequencing-grade trypsin (Promega, Madison, WI) in a 1:30 (*w/w*) trypsin:protein ratio and desalted by solid-phase extraction using 1cc HLB cartridges (Waters, MA, USA), as previously described [3].

Desalted peptides (300 ng) were analysed using a Q-Exactive^TM^ HFX3 hybrid quadrupole-orbitrap mass spectrometer (Thermo Scientific, MA, USA). Peptide mixtures (0.1 μg/μl) resuspended in 3% (v/v) ACN/0.1% (v/v) FA were separated by nano-LC using an Ultimate 3000 UHPLC and autosampler system (Dionex, Amsterdam, Netherlands). Reverse-phase mobile buffers were composed of A: 0.1% (v/v) FA (Thermo Scientific, MA, US, Cat No. 34851-4), and B: 80% (v/v) ACN (Thermo OPTIMA LC-MS grade, Cat No. 34851-4) /0.1% FA. Peptides were eluted using a linear gradient of 5% B to 42% B across 140 min with a constant flow rate of 250 nL min-1. High voltage (2000 V) was applied to a low volume tee (Valco, Houston, TX, USA) and the column tip positioned approximately 0.5 cm from the heated capillary (T = 275 °C) of the MS. Positive ions were generated by electrospray and the Orbitrap was operated in data-independent acquisition (DIA) mode. A total of 20 variably sized windows (including 1.0 Da window overlap) were generated covering a precursor mass range of 350–1650 m/z. While m/z ratios selected for MS/MS were dynamically excluded for 20 s. Prior to loading the samples, a LC-MS/MS standard consisting of 30 fmol pre-digested BSA (GeneSearch, QLD, Australia, Cat No. P8108S, 500 pmol) was injected to ensure optimal performance and dynamic range of the instrument. This was repeated throughout the analysis, along with one patient sample, to ensure technical reliability and sensitivity of the instrument was maintained.

### Analysis of DIA LC-MS/MS data

A comprehensive GBM spectral library previously generated from primary patient-derived GBM cells, GBM-EVs enriched from surgical fluids and GBM tumour tissues [5] was used for targeted DIA-MS data extraction of uEV proteomes. The library contained spectral data for 8651 protein groups and 186037 precursors. The DIA-MS data was aligned and searched against the spectral library using data independent neural network (DIA-NN1.8^TM^) with the following parameters: Trypsin, two missed cleavages; maximum number of variable modifications, 1; variable peptide modifications, M excision, carbamidomethylation and oxidation; peptide length range, 7–30; precursor m/z range, 300–1800; fragment ion m/z range, 100–2000; precursor false discovery rate, 1%; quantification strategy, robust LC (high accuracy). The mass accuracy was optimised by DIA-NN1.8 and scan windows were inferred separately for each data sample.

After removing low confidence identifications and interfering precursors, DIA-NN allowed a MaxLFQ-based protein quantification of the proteins identified at 1% false discovery rate (FDR) [33]. The MaxLFQ abundance values for identified proteins was output into quantities matrices. The repeatability and reproducibility of the DIA-MS approach was assessed by correlating the abundances of our replicate MS injections of the same uEV sample (UH10-EV, Int Ctrl) captured at different timepoints during the data acquisition. The goodness-of-fit measure for linear regression (coefficient of determination, R^2^) found high reproducibility and repeatability of the replicate injections; 0.9423 < R^2^ < 0.9811 Supplementary Fig. 2). The raw mass spectrometry proteomics data, spectral library and results have been deposited to the ProteomeXchange Consortium via the PRIDE [34] partner repository with the dataset identifier PXD046511.

### DIA-MS data filtering, normalisation, statistics, and visualisation

The DIA-MS uEV data was filtered, processed, and normalised with Perseus 1.6.5.0 [34]. A total of 903 proteins were selected for further differential expression analysis as they were identified in at least 80% of samples in all cohorts (Pre-OP, Post-OP, REC, HC). The proteomes of each cohort were annotated to Vesiclepedia, sites of expression, cellular components, and biological pathways using FunRich 3.1.3 [35]. The protein abundance levels were processed by a log2 transformation, followed by an imputation of missing values from the normal distribution, and quantile normalisation to adjust for differences in sample size and measurement bias. Normalised data was used to calculate statistical differences between groups using START: Shiny Transcriptome Analysis Resource Tool/2019/ (https://kcvi.shinyapps.io/START/) [36] and unpaired Student’s *t*-test. Differentially abundant proteins between the GBM vs HC and Pre-OP vs REC with FC ≥ | 2| and *p*-adjust≤0.05 were subjected to ROC curve analysis and simple logistic regression (GraphPad Prism). Proteins with Area under the ROC Curve (AUC) > 0.9 were selected for further analysis to determine biomarker performance. Stepwise logistic regression models were constructed using selected proteins (FC ≥ | 2 | , adjust *p*-value ≤ 0.05, AUC > 0.9) to determine the best performing panel of proteins in the ‘diagnostic’ (GBM vs HC) and ‘progression’ (Post-OP vs REC) signatures. Here, the Logit score was calculated using the following equation: ${\sum }_{i=1}^{n}{expi}* {\beta }_{i}$, where *n* = number of prognostic proteins; expi=expression level of prognostic protein I; βi is the regression coefficient of protein i. The logit score was then used to generate a ROC analysis for the regression model. By leaving out one protein at a time from the logit score calculation, the number and combination of proteins with the best biomarker performance was determined. Differentially abundant proteins were visualised as volcano plots and box plots, and principal component analysis (PCA) was used to visualise the protein signature performance in separating the sample groups. Results were visualised using GraphPad Prism (San Diego, California) and figures prepared in Adobe Illustrator (San Jose, CA).

### Functional annotations of identified proteins

Pathway analysis was performed using Ingenuity® software (Ingenuity Systems, USA; http://analysis.ingenuity.com) to assess functional associations (biological and canonical pathways) of differentially abundant proteins (*p* ≤ 0.05) by performing core expression analyses using default criteria. TRiC subunit proteins (TCP1, CCT2, CCT3, CCT4, CCT5, CCT6A, CCT6B, CCT7, CCT8) and interacting partners in the GBM vs HC dataset were explored using the grow and overlay functions in the pathway designer. KEGG and Reactome Pathways were annotated for the TRiC interactome of 42 proteins using The Database for Annotation, Visualisation, and Integrated Discovery (DAVID). Listed pathways had a fold-enrichment >2 and *p*-value < 0.05.

## Results

### Characterisation of uEVs and the GBM uEV proteome

**Fig. 1:**
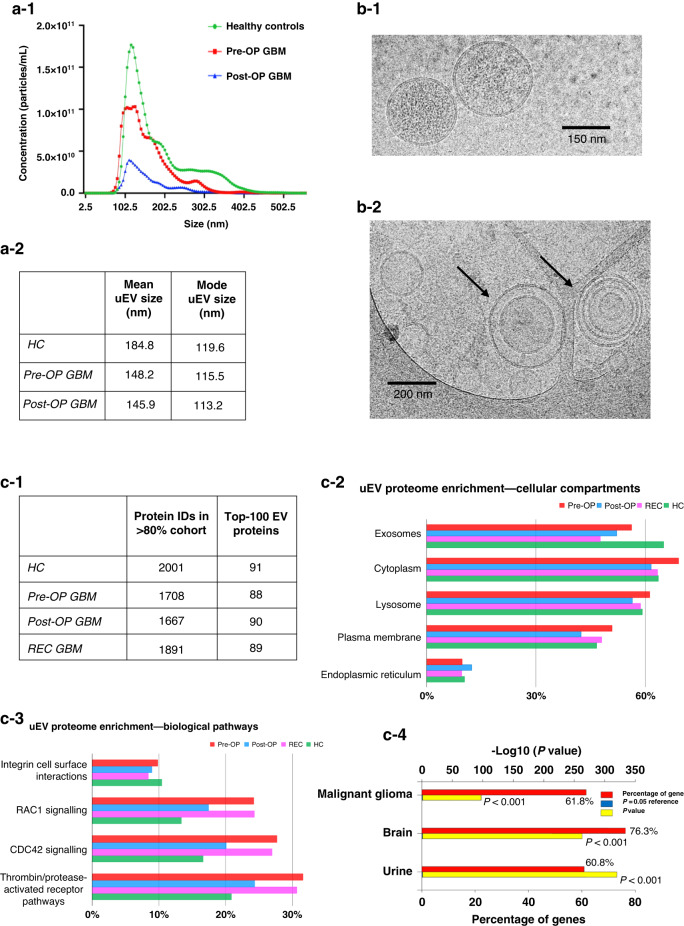
Characterisation of urinary-EVs (uEVs). Nanoparticle tracking analysis (NTA) determined **(a-1)** the concentration (particles per mL) and size distributions of uEVs captured from healthy controls (HC, *n* = 3), Pre-OP (*n* = 3) and Post-OP GBM patients (*n* = 3). **a-2** The mean and modal EV sizes of each group are tabulated. **b-1** Cryo-transmission electron microscopy (cryo-TEM) images of uEVs from a healthy individual showed characteristic vesicular morphology as well as **(b-2)** evidence of heterogenous particle morphology, including EVs with multiple membranes (arrows; scale bar = 100 nm). **c-1** Liquid chromatography coupled tandem mass spectrometry (LC-MS/MS) identified 1667-2001 protein species in >80% of samples in each cohort (Pre-OP, Post-OP, GBM and HC), that included 88-91% of the top-100 EV proteins (*as reported by Vesiclepedia*). **c-2** FunRich analysis of GBM Pre-OP uEV specimens revealed significant enrichments of proteins annotated to exosome related cellular compartments and (**c-3**) biological pathways associated with cancer-related processes. **c-4** GBM uEV proteomes are associated with highly relevant sites of expression, i.e., ‘malignant glioma’, ‘brain’ and ‘urine’ (*p* < 0.001).

**Fig. 2:**
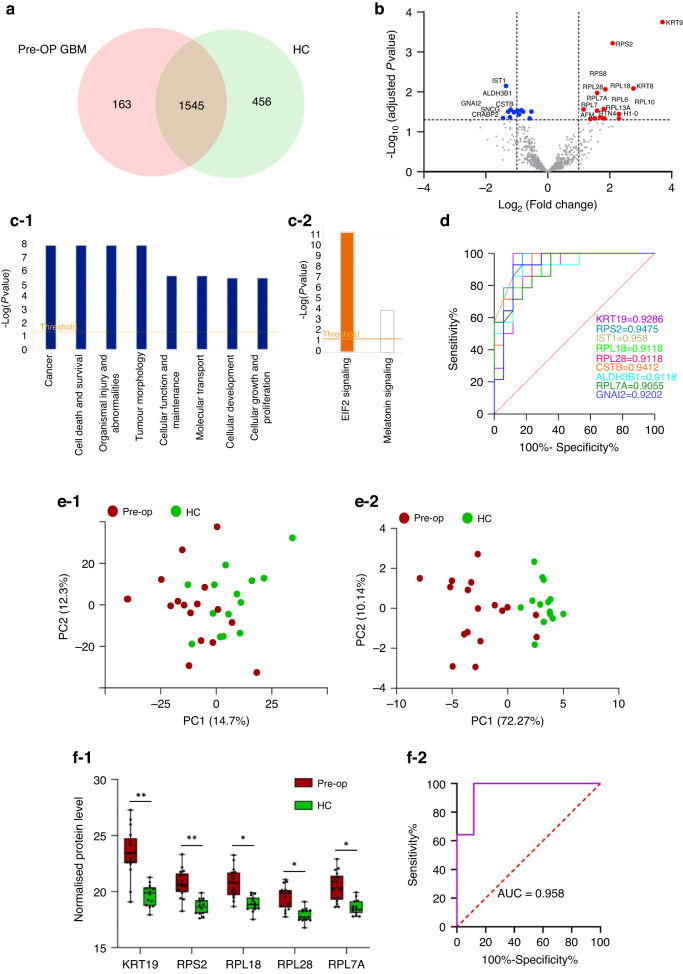
uEV proteins show promise as diagnostic biomarkers for GBM. **a** Venn diagram of proteins confidently identified in >80% of samples in Pre-OP GBM (*n* = 16; *1708 proteins*) and HC (*n* = 14; *2001 proteins*) groups shows an overlap of 1545 proteins. **b** Volcano plot depicting the differentially abundant proteins between GBM Pre-OP and HC groups; proteins with increased (red) and decreased (blue) levels in GBM Pre-OP relative to HC (adjusted *p*-val≤0.05 cut-off); proteins with FC ≥ | 2| are labelled with their gene names. **c-1** Ingenuity Pathway Analysis of the differentially abundant proteins (FC ≥ | 2 | , *p*-val≤0.05) revealed significant associations to disease and functional pathways related to cancer, and **(c-2)** the predicted activation of ‘*EIF2 signaling*’ (z-score: 2.646; *p*-value: 5.35e^-12^). **d** Nine significant proteins (KRT19, RPS2, IST1, RPL18, RPL28, CSTB, ALDH3B1, RPL7A, GNAI2; FC ≥ | 2| and adjust *p*-val≤0.05) show excellent sensitivity and specificity with AUC values > 0.9. Principal component analysis (PCA) plots showing **(e-1)** minimal discrimination of Pre-OP GBM and HC samples based on the levels of the 1545 common proteins and **(e-2)** improved discrimination of Pre-OP GBM and HC samples based on the nine proteins with AUC > 0.9, FC ≥ | 2| and adjust *p*-val≤0.05. **f-1** A stepwise logistic regression model revealed a panel of five putative diagnostic uEV proteins (KRT19, RPS2, RPL18, RPL28, RPL7A) **(f-2)** with a cumulative AUC performance of 95.8%. Significance levels are denoted by *adjust *p*-val≤0.05, ** adjust *p*-val≤0.001.

uEVs were isolated by differential ultracentrifugation and characterised by nanoparticle tracking analysis (NTA) with average EV size distributions and concentrations determined from biological and technical triplicate readings. NTA revealed that the uEV isolates were enriched with small-EV populations of similar size distributions ( < 300 nm; Fig. 1a-1). uEVs from healthy controls (HCs, *n* = 3) had a significantly higher mean uEV size relative to GBM patients (pre-operative (Pre-OP), *n* = 3; post-operative (Post-OP), *n* = 3), however modal EV sizes were similar between the sample groups (Fig. 1a-2; Supplementary Fig. 3). Cryo-transmission electron microscopy (cryo-TEM) imaged vesicles of ranging sizes, predominantly between 80–150 nm (Fig. 1b-1), with some morphological heterogeneity observed including nanoparticles with single, double, and quadruple membranes (*arrows*; Fig. 1b-2). Further EV characterisation was performed by analysing isolated uEV proteomes prepared from GBM patients and HCs by DIA-MS (Fig. 1c-1).

A total of 6857 unique protein species were confidently identified across all uEV specimens (*q*-value ≤ 0.01), including 94 of the top-100 EV marker proteins curated by Vesiclepedia (Supplementary Table 2). Canonical EV marker proteins identified in all uEVs included programmed cell death 6-interacting protein (PDCD6IP), CD9, CD63 and tumour susceptibility gene 101 protein (ESCRT-I complex subunit TSG101). No identified uEV proteins were exclusively detected in either the GBM or HC cohorts. A total of 1708, 1667, 1891 and 2001 proteins were identified in more than 80% of specimens in Pre-OP, Post-OP, recurrent (REC) and HC cohorts, respectively (Fig. 1c-1; Supplementary Table 3). Overall, 2195 proteins were identified across the GBM uEV specimens (GBM Pre-OP, Post-OP and REC). A total of 903 proteins were identified in >80% of samples across all uEV cohorts (Pre-OP, Post-OP, REC and HC), and were selected for differential expression analysis (Supplementary Tables 4 and 5).

**Fig. 3:**
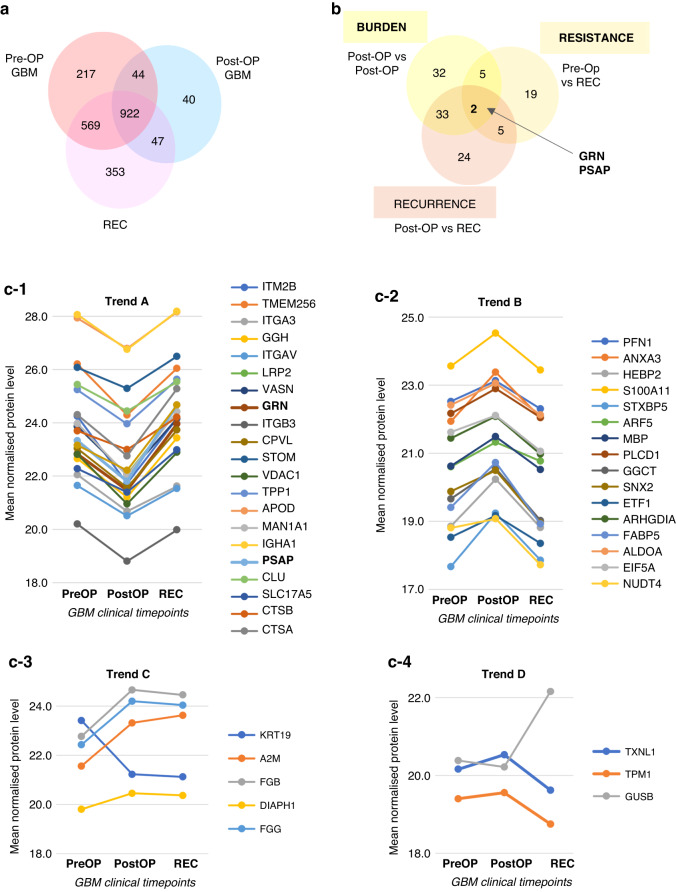
Significant uEV protein changes corresponding to different GBM clinical timepoints. Venn diagrams depicting overlapping (**a**) protein species confidently identified in GBM uEVs captured before primary surgery (Pre-OP), after primary surgery (Post-OP) and before GBM recurrence (REC) surgery, and (**b**) differentially abundant protein changes from pairwise comparisons (*p*-val≤0.05). Mean normalised protein levels were plotted over the three GBM clinical timepoints and grouped according to four observed trends. **c-1** Trend A describes proteins whose levels are significantly reduced Post-OP and return to Pre-OP levels at REC (*p*-val≤0.05), and **(c-2)** Trend B describes protein species whose levels are significantly increased Post-OP and return to Pre-OP levels at REC (*p*-val≤0.05). **c-3** Trend C includes proteins that change significantly Post-OP and remain steady at REC (*p*-val≤0.05) and **(c-4)** Trend D includes proteins that significantly change only at REC (*p*-val≤0.05).

**Fig. 4:**
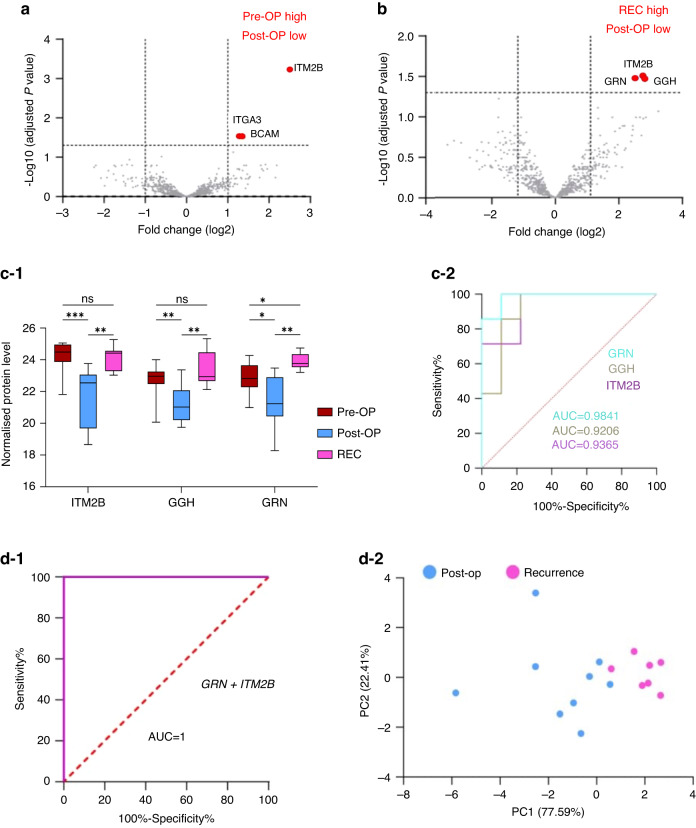
GBM uEV biomarker selection and performance. Volcano plot of significant uEV proteins changes corresponding to (**a**) GBM tumour burden (Pre-OP vs Post-OP) and (**b**) GBM recurrence (Post-OP vs REC); proteins changing at FC ≥ | 2| and adjust *p*-val≤0.05 are labelled with their corresponding gene names. **c-1** Longitudinal normalised abundances of ‘GBM recurrence proteins’, ITM2B, GGH and GRN, follow the same trend across the Pre-OP, Post-OP and REC samples (*p ≤ 0.05, **adjust *p*-val≤0.05, ***adjust *p*-val≤0.001, and ‘*ns*‘ non-significant change) and **(c-2)** show excellent individual performance in receiver operator characteristic (ROC) analyses (AUC > 0.92). **d-1** Stepwise logistic regression revealed that when considered together, GRN and ITM2B protein levels in uEVs are highly sensitive biomarkers for GBM recurrence with a combined AUC = 1.0 **d-2** and clear separation of Post-OP and REC samples on PCA plot.

Functional enrichment analysis showed that resolved uEV proteomes were significantly annotated to cellular compartments associated with EVs, i.e., ‘exosomes’, ‘lysosomes’, and ‘plasma membrane’ (Fig. 1c-2). Potential contamination of the uEV preparations with endoplasmic reticulum (i.e., calreticulin, protein disulfide isomerases) and golgi apparatus proteins was observed, however the cis-Golgi matrix protein GM130 was not sequenced (Supplementary Table 2). uEV proteomes were significantly annotated to biological pathways with important roles in various tumour progression processes (Fig. 1c-3). Of note, the proteomes of Pre-OP and REC samples are more highly annotated with proteins involved in RAC1 signalling, CDC42 signalling, and the thrombin/protease activated receptor pathway than Post-OP and HC samples (Fig. 1c-3). Encouragingly, GBM uEV proteomes were significantly annotated to relevant expression sites, including ‘malignant glioma’, ‘brain’ and ‘urine’ (*p* ≤ 0.001; Fig. 1c-4).

### Urinary EV proteins show promise as GBM diagnostic biomarkers

Overall, 1545 proteins were common between Pre-OP primary GBM samples and HC (Fig. 2a). Of these, 209 and 31 proteins were identified to change between the two groups at *p*-value ≤ 0.05 and Benjamini Hochberg (BH)-adjusted *p*-value ≤ 0.05 thresholds, respectively (Supplementary Table 5; Fig. 2b), and may indicate the presence of a GBM tumour. Ingenuity Pathway Analysis (IPA) revealed significant associations to disease and functional annotations, including all 31 molecules associated with ‘*cancer’* and ‘*tumour morphology’* (Fig. 2c-1) and the predicted activation of canonical pathway, ‘*EIF2 signaling*’ (z-score: 2.646; *p*-value: 5.35e^-12^; Fig. 2c-2).

Nine of the 31 proteins (*ALDH3B1, CSTB, GNAI2, IST1, KRT19, RPS2, RPL7A, RPL18, RPL28;* FC ≥ 2 in GBM Pre-OP relative to HC, adjusted *p*-val≤0.05) showed excellent diagnostic sensitivity and specificity on receiver operating characteristic (ROC) analysis (area-under-the ROC curve, AUC > 0.9; Fig. 2d) and improved the separation of Pre-OP GBM and HC specimens in a principal component analysis compared to the 1545 shared proteins (Fig. 2e-1, e-2). A stepwise logistic regression further refined this list to five putative uEV biomarker proteins (*KRT19, RPS2, RPL18, RPL28, RPL7A*) that had significantly higher levels in GBM Pre-OP samples relative to HC (Fig. 2f-1), with an excellent accumulative diagnostic performance of 95.8% (AUC = 0.958; Fig. 2f-2).

### Descriptive EV-urinary proteome changes corresponding to different GBM clinical timepoints

Pairwise comparisons were performed between the three GBM cohorts (Pre-OP, Post-OP and REC) to determine biomarker proteins corresponding to different aspects of the GBM clinical timeline. First, a GBM ‘tumour burden’ proteome signature was identified by comparing the differential abundance of Pre-OP and Post-OP GBM uEV proteins. A total of 966 proteins were common to GBM Pre-OP and Post-OP uEVs (Fig. 3a), and levels of 72 proteins were identified to change significantly in uEVs following the total gross resection of primary GBM tumours (GBM ‘tumour burden’ proteins; *p* ≤ 0.05; Fig. 3b, Supplementary Table 5), including three proteins (BCAM, ITGA3, ITM2B) reduced by more than 2-fold in Post-OP relative to Pre-OP samples (adjust *p*-value ≤ 0.05; see Fig. 4a). Of the 72 GBM ‘tumour burden’ proteins, 20 were also significantly associated with a GBM diagnosis (i.e., 20 proteins also changed significantly between GBM Pre-OP versus HC; *p*-val≤0.05; Supplementary Table 5). Interestingly, all 20 proteins showed the same direction of change between high-tumour burden samples (GBM Pre-OP) and low-tumour burden/non-tumour samples (GBM Post-OP and HC), indicating that after the surgical removal of a primary tumour, these GBM diagnostic proteins are at levels similar in HCs (Supplementary Fig. 4).

Next, we explored uEV proteome changes associated with GBM ‘tumour recurrence’ by comparing protein levels between the Post-OP and REC cohorts. A total of 969 proteins were common to Post-OP and REC uEVs (Fig. 3a). Relative to Post-OP levels, 64 uEV proteins changed significantly at GBM tumour recurrence (*p* ≤ 0.05; Fig. 3b, Supplementary Table 5), including three proteins (GGH, GRN, ITM2B) that increased by ≥2-fold in the REC group (adjust *p*-val≤0.05; Fig. 4b). Lastly, uEV proteome changes associated with GBM ‘treatment resistance’ were investigated by comparing patients with primary and recurrent GBM (pre-OP versus REC). A total of 1491 proteins were common to the Pre-OP and REC uEVs (Fig. 3a) and 31 proteins were identified to be significantly associated with ‘treatment resistance’ (*p* ≤ 0.05; Fig. 3b, Supplementary Table 5), however none of these uEV protein changes met the more stringent significance threshold of an adjusted *p*-val ≤0.05.

Aligning the significantly changing proteins (*p* ≤ 0.05) across the three comparative analyses (GBM tumour ‘burden’, ‘recurrence’ and ‘resistance’) revealed some interesting overlapping trends across the GBM clinical timepoints (Pre-OP, Post-OP and REC; Fig. 3b). Of note, 45 proteins were differentially abundant across the three comparisons, with GRN and PSAP proteins common to all analyses, and showed excellent discrimination of uEV specimens from the three different GBM cohorts (Supplementary Fig. 5). Of the 45 intersecting protein changes, the majority (35 proteins, 78%) were shared between the ‘burden’ and ‘recurrence’ comparisons and followed similar trends of change, i.e., abundance levels significantly changed post-operatively but returned to Pre-OP primary GBM levels at recurrence (Fig. 3c-1, c-2). These protein changes may relate to the surgical procedure itself or indeed reflect common GBM markers associated with both primary and recurrent tumours. The remaining proteins appear to reflect changes specific to the presence of a primary GBM (Fig. 3c-3) or treatment-resistant GBM recurrence (Fig. 3c-4). Overall, 64 putative GBM biomarker proteins identified in uEVs here were also previously described in GBM-EVs isolated from other biofluids (See Table 2 for summary).

**Table 2:** Summary of significantly changing uEV proteins previously identified as GBM-EV biomarkers in other biofluids (ex vivo CUSA or neurosurgical aspirates, peripheral plasma and GBM culture media).

Uniprot ID	Gene Name	Protein Name	GBM uEV Diagnostic	GBM uEV Burden	GBM uEV Recurrence	GBM uEV Resistance	Significant GBM-EV finding in other biofluid	REF
P31949	S100A11	Protein S100-A11	**X**	X	X		**CUSA-EV**	[3]
P07602	PSAP	Prosaposin		X	X	X	**PLASMA-EV** + in vitro **marker**	[5, 40]
Q14764	MVP	Major vault protein	X		X		in vitro **marker**	[40]
Q9NZM1	MYOF	Myoferlin		X	X		**CUSA-EV**	[3]
Q01469	FABP5	Fatty acid-binding protein 5		X	X		**CUSA-EV**	[3]
Q92820	GGH	Gamma-glutamyl hydrolase		X	X		in vitro **EV marker**	[40]
P21796	VDAC1	Voltage-dependent anion-selective channel protein 1		X	X		**CUSA-EV**	[3]
P04075	ALDOA	Fructose-bisphosphate aldolase A		X	X		in vitro **EV marker**	[40]
P10619	CTSA	Lysosomal protective protein		X	X		in vitro **EV marker**	[40]
P10909	CLU	Clusterin		X	X		in vitro **EV marker**	[40]
P01023	A2M	Alpha-2-macroglobulin		X		X	in vitro **EV marker**	[40]
P04080	CSTB	Cystatin-B	X				**CUSA-EV**	[3]
Q5VW32	BROX	BRO1 domain-containing protein BROX	X				**PLASMA-EV**	[5]
P13987	CD59	CD59 glycoprotein	X				**CUSA-EV**	[3]
P25786	PSMA1	Proteasome subunit alpha type 1	X				in vitro **EV marker**	[40]
Q9UN37	VPS4A	Vacuolar protein sorting-associated protein 4A	X				**PLASMA-EV**	[5]
Q13200	PSMD2	26 S proteasome non-ATPase regulatory subunit 2	X				**CUSA EV** + in vitro **marker**	[3, 40]
O00231	PSMD11	26 S proteasome non-ATPase regulatory subunit 11	X				in vitro **EV marker**	[40]
P35579	MYH9	Myosin-9	X				in vitro **EV marker**	[40]
P46781	RPS9	40 S ribosomal protein S9	X				**CUSA EV** + In vitro **marker**	[3, 40]
P22695	UQCRC2	Cytochrome b-c1 complex subunit 2, mitochondrial	X				**CUSA-EV**	[3]
Q5JWF2	GNAS	Guanine nucleotide-binding protein G(s) subunit alpha isoforms XLas	X				**CUSA-EV**	[3]
P07355	ANXA2	Annexin A2	X				**CUSA-EV**	[3]
P04083	ANXA1	Annexin A1	X				**CUSA EV** + In vitro **marker**	[3, 40]
O43242	PSMD3	26 S proteasome non-ATPase regulatory subunit 3	X				in vitro **EV marker**	[40]
P49368	CCT3	T-complex protein 1 subunit gamma	X				**CUSA EV** + In vitro **marker**	[3, 40]
P55072	VCP	Transitional endoplasmic reticulum ATPase	X				in vitro **GBM EV marker**	[40]
Q16851	UGP2	UTP--glucose-1-phosphate uridylyltransferase	X				**CUSA EV** + In vitro **marker**	[3, 40]
P53396	ACLY	ATP-citrate synthase	X				in vitro **EV marker**	[40]
P52907	CAPZA1	F-actin-capping protein subunit alpha-1	X				**CUSA-EV**	[3]
Q99832	CCT7	T-complex protein 1 subunit eta	X				**PLASMA-EV** + **CUSA EV**	[3, 5]
P49327	FASN	Fatty acid synthase	X				in vitro **EV marker**	[40]
O75882	ATRN	Attractin	X				in vitro **EV marker**	[40]
P59666	DEFA3	Neutrophil defensin 3	X				**CUSA-EV**	[3]
P55060	CSE1L	Exportin-2	X				**PLASMA-EV**	[4]
Q14974	KPNB1	Importin subunit beta 1	X				in vitro **EV marker**	[40]
P17987	TCP1	T-complex protein 1 subunit alpha	X				**CUSA EV** + In vitro **marker**	[3, 40]
Q86VP6	CAND1	Cullin-associated NEDD8-dissociated protein 1	X				in vitro **EV marker**	[40]
P01008	SERPINC1	Antithrombin-III	X				in vitro **EV marker**	[40]
P40227	CCT6A	T-complex protein 1 subunit zeta	X				**CUSA-EV**	[3]
P28838	LAP3	Cytosol aminopeptidase	X				**CUSA-EV**	[3]
P60174	TPI1	Triosephosphate isomerase	X				in vitro **EV marker**	[40]
Q92542	NCSTN	Nicastrin	X				**CUSA-EV**	[3]
P50991	CCT4	T-complex protein 1 subunit delta	X				in vitro **EV marker**	[40]
O15498	YKT6	Synaptobrevin homolog YKT6	X				**PLASMA-EV**	[5]
O60884	DNAJA2	DnaJ homolog subfamily A member 2	X				**CUSA-EV**	[3]
P01011	SERPINA3	Alpha-1-antichymotrypsin	X				**CUSA-EV**	[3]
P25705	ATP5F1A	ATP synthase subunit alpha, mitochondrial	X				**CUSA-EV**	[3]
Q9UBI6	GNG12	Guanine nucleotide-binding protein G(I)/G(S)/G(O) subunit gamma-12	X				**CUSA-EV**	[3]
P08238	HSP90AB1	Heat shock protein HSP 90-beta	X				**CUSA EV** + In vitro **marker**	[3, 40]
P07900	HSP90AA1	Heat shock protein HSP 90-alpha	X				**CUSA EV** + In vitro **marker**	[3, 40]
P62258	YWHAE	14-3-3 protein epsilon	X				in vitro **EV marker**	[40]
O00299	CLIC1	Chloride intracellular channel protein 1	X				**CUSA-EV**	[3]
P45880	VDAC2	Voltage-dependent anion-selective channel protein 2		X			**CUSA-EV**	[3]
P05362	ICAM1	Intercellular adhesion molecule 1 (ICAM-1)		X			**CUSA-EV**	[3]
P06727	APOA4	Apolipoprotein A-IV		X			**CUSA-EV**	[3]
P05556	ITGB1	Integrin beta 1		X			**PLASMA-EV** + in vitro **marker**	[5, 40]
P07339	CTSD	Cathepsin D			X		in vitro **EV marker**	[40]
Q9NT62	ATG3	Ubiquitin-like-conjugating enzyme ATG3			X		**CUSA-EV**	[3]
P01009	SERPINA1	Alpha-1-antitrypsin			X		**CUSA-EV**	[3]
O75369	FLNB	Filamin-B			X		in vitro **EV marker**	[40]
P28074	PSMB5	Proteasome subunit beta type 5			X		in vitro **EV marker**	[40]
Q99497	PARK7	Parkinson disease protein 7				X	**PLASMA-EV**	[5]
P20618	PSMB1	Proteasome subunit beta type 1				X	in vitro **EV marker**	[40]

### GBM recurrence biomarker selection and performance

The performance of the three putative GBM recurrence biomarkers (GGH, GRN and ITM2B; Fig. 4b, c-1) were assessed by ROC analyses, each revealing excellent sensitivity and specificity in distinguishing Post-OP from REC specimens with AUC > 0.92 (Fig. 4c-2). A stepwise logistic model assessed the cumulative performance of these three uEV proteins, and selected ITM2B and GRN as the best performing biomarkers with a combined AUC = 1.0 (Fig. 4d-1). Together, GRN and ITM2B showed clear separation of Post-OP and REC samples when visualised on a PCA plot (Fig. 4d-2).

### The T-complex protein ring complex (TRiC) interactome of GBM uEVs

**Fig. 5:**
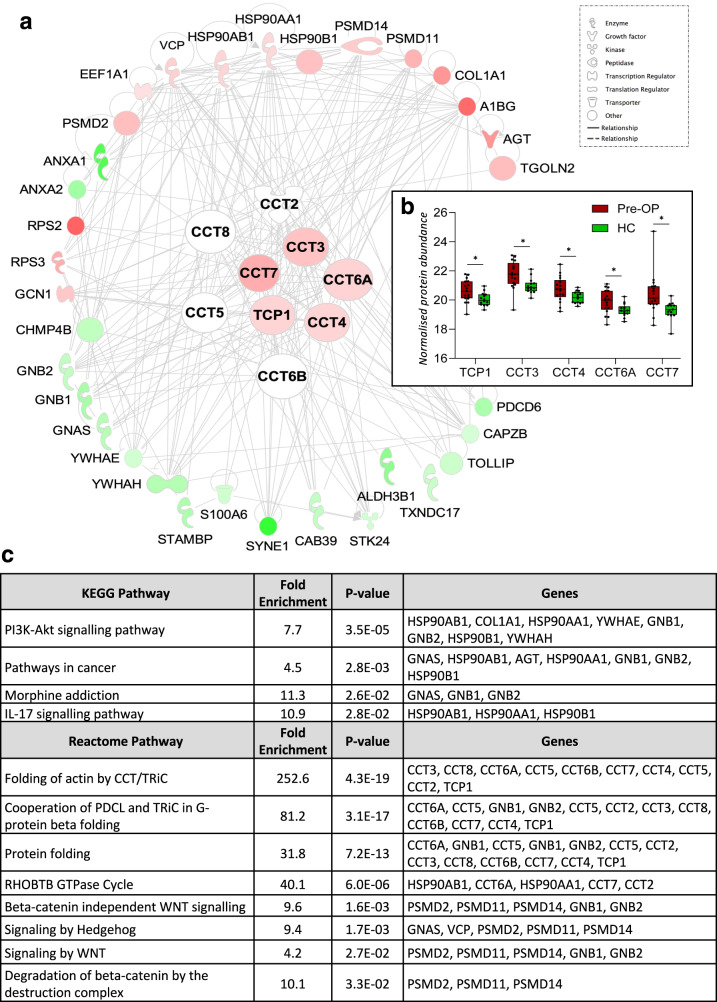
Functional analysis exploring the interactions of TRiC proteins and differentially abundant proteins between GBM Pre-OP and HC uEVs. **a** Ingenuity Pathway Analysis network for TRiC proteins and their interacting partners. The network was generated from differentially abundant proteins between GBM Pre-OP and HC uEVs. Proteins with higher and lower expression in GBM Pre-OP relative to HC are *red* and *green*, respectively. Darker shades depict higher fold-changes. **b** TRiC subunit changes presented in boxplots, *p-value < 0.05. **c** DAVID functional annotations of the 42 TRiC interactome proteins for KEGG and Reactome Pathways. Significant pathway annotations are listed that have a fold enrichment ≥2 and *p*-value < 0.05.

Interestingly, all eight T-complex protein Ring Complex (TRiC) subunits, TCP1, CCT2, CCT3, CCT4, CCT5, CCT6A, CCT7 and CCT8, were identified in GBM uEVs (Supplementary Table 3). Five of the subunits (TCP1, CCT3, CCT4, CCT6A and CCT7) are putative GBM uEV diagnostic proteins with significantly higher levels in GBM Pre-OP uEVs compared to HC (FC ≥ 2, *p*-val≤0.05; Fig. 5b; Supplementary Table 5) and were previously reported in GBM-EVs derived in vitro cells, from neurosurgical aspirates (CUSA) and plasma (Table 2). Using IPA, TRiC proteins (TCP1, CCT3, CCT4, CCT6A and CCT7) and their interacting partners were explored in the set of differentially abundant proteins between GBM and HC uEVs. IPA resolved 33 TRiC subunit interacting protein partners, including GBM uEV diagnostic proteins ANXA1, ANXA2, GNAS, HSP90AA1, HSP90AB1, PSMD2, PSMD11, VCP and YWHAE (Fig. 5a, Table 2), as well as other TRiC subunits, CCT2, CCT5 and CCT8. Next, the TRiC protein subunits and 33 interacting proteins were annotated to the Kyoto Encyclopedia of Genes and Genomes (KEGG) and Reactome Pathways by The Database for Annotation, Visualisation and Integrated Discovery (DAVID) and revealed significant enrichments in ‘pathways in cancer’ (4.5-fold, *p*-val = 0.0028), ‘PI3K/Akt signaling pathway’ (7.7-fold, *p*-val = 0.000035), ‘signaling by WNT’ (4.2-fold, *p*-val = 0.027), ‘Beta-catenin independent WNT signaling’ (9.6-fold, *p*-val = 0.016) and ‘signaling by Hedgehog’ (9.4-fold, *p*-val = 0.017; Fig. 5c).

## Discussion

### uEVs as a novel source of GBM biomarkers

New, minimally invasive tumour surveillance approaches that can sensitively detect tumour changes are vital to improving clinical care and outcomes for GBM patients. In this feasibility study, uEVs were investigated as a novel source of dynamic GBM biomarkers and we present evidence supporting the development of ‘*liquid gold’* biopsies to monitor tumour progression and treatment resistance. EV-associated GBM proteomic signatures have been described in neurosurgical ‘CUSA’ washings, CSF, and peripheral blood [4–6]. However, as substrates for routine liquid biopsies, surgical fluid and CSF are limited by highly invasive collection procedures, and the enormously complex and dynamic molecular constituents of the blood has challenged biomarker discovery [37]. As a liquid biopsy biofluid, the urine offers a practicable alternative that can be non-invasively and frequently sampled in large volumes. Urine is less complex than blood [38], and uEV protein biomarkers are extremely stable with minimal impacts on analytical findings observed when unfractionated urine is stored for up to 3 days at 4 °C, and 24 h at 20 °C [39]. The urine also accumulates systemic changes and as it is the major route of EV clearance, is an abundant source of EVs [20].

Arguably, robust GBM-EV biomarkers are those that can be detected in various body fluids, allowing the development of flexible GBM diagnostic assays that can use multiple body fluids. Of the many interesting proteins resolved, A2M, ALDOA, CLU, CTSA, FABP5, GGH, MVP, MYOF, S100-A11, PSAP, VDAC1 warrant specific mention as they were identified in at least two comparative analyses here and were previously identified as GBM-EV biomarkers in other biofluids (Table 2) [3, 5, 40]. Additionally, ALDOA was recently identified in salivary EVs from GBM patients [41]. However, EVs also harbour distinct molecular repertoires in different body compartments, particularly at their surface, perhaps in part due to the highly changeable EV coronal layer and interactome that is influenced by the changing molecular environment [42]. Therefore, differences in GBM-EV biomarkers across various body fluids need to be accounted for during the conceptualisation of EV-based diagnostic assays. It is also important to note that total uEV populations were assessed in this study as opposed to fractionated EV populations captured from other biofluids [3, 5, 40].

### uEV proteome corresponds to a GBM diagnosis

Fundamentally, uEV proteomes can readily distinguish GBM patients from healthy controls. Despite the caveats above, the detection of uEV proteins previously reported as GBM biomarkers is encouraging (Table 2) as are the significant functional annotations to ‘cancer’ and ‘tumour morphology’ (Fig. 2c-1). Here, IPA predicted the significant activation of EIF2 signalling as a downstream consequence of GBM diagnostic uEV protein levels (*p*-val = 5.35e^−12^; Fig. 2c-2). Indeed, EIF2 signalling activation was previously observed in vitro following the exposure of non-neoplastic astrocytes with GBM-derived EVs [43], supporting the notion that a GBM signal is detectable in uEVs.

Around 20% (44/209) of uEV proteins changing between Pre-OP GBM and HC (*p* ≤ 0.05) were previously identified as potential GBM-EV biomarkers (Table 2). Of note, eight T-Complex protein 1-Ring complex (TRiC) complex subunits (TCP1, CCT2, CCT3, CCT4, CCT6A, CCT7 and CCT8) were identified in uEVs, and five subunits (TCP1, CCT3, CCT4, CCT6A and CCT7) were significantly higher in GBM Pre-OP uEVs relative to HC. This finding recapitulates our observations in neurosurgical EVs where all eight TRiC protein subunits were significantly higher in IDHwt GBM patients relative to IDH-mutant glioma grade 2 to 3 patients, and TRiC gene levels were higher in GBM tissue relative to normal brain [3]. We previously reported CCT6A as a negative survival marker for GBM, its co-localisation with EGFR at 7p11.2 with a strong tendency for gene co-amplification and protein expression [3]. Similarly, TRiC subunits CCT2, CCT3, CCT4, CCT5, CCT7 and TCP1, were measured in higher levels in the plasma of GBM patients relative to healthy controls, with significant increases in CCT2 and CCT7 [5]. Another study later corroborated that high CCT6A levels in glioma correlate with elevated WHO grade, fewer *IDH1/2* mutations and shorter overall survival [44].

There is also considerable overlap between TRiC interactome proteins identified in GBM uEVs and our previously published studies of EVs derived from GBM neurosurgical aspirates (Table 2, Fig. 5a). Of note, ANXA1, ANXA2, GNAS, HSP90AA1, HSP90AB1 and PSMD2, were highly abundant in EVs captured from GBM neurosurgical aspirates and in Pre-OP GBM uEVs here, implicating a potential brain tumour diagnostic utility for TRiC subunits and their interacting partners [3]. Typically, abundant TRiC levels are associated with rapidly dividing cells [45] and are implicated in oncogenesis through Wnt/β-catenin and PI3K/AKT pathway activations, both key signalling axes in GBM [46]. Indeed, TRiC subunits are significantly associated with aggressive cancer traits and poor outcomes in hepatocellular carcinoma [47], Ewing sarcoma [48], breast cancer [49, 50], neuroblastoma [51], gastric cancer [52], and lung adenocarcinoma [53].

Intriguingly, multiple ribosomal protein subunits were also among the GBM diagnostic proteins (RPS2, RPL18, RPL28, RPL7, RPL6, RPS8, RPL7A, RPL10, RPL13A), all of which were significantly higher in GBM uEVs relative to HC (Fig. 2b). Ribosome biogenesis and protein synthesis are fundamental rate-limiting steps for cell growth and proliferation and are essential characteristics that enable cancer cells to sustain uncontrolled proliferation. Emerging evidence suggests that cancer cells harbor a specialised class of ribosomes (onco-ribosomes) that facilitates the oncogenic translation program, modulates cellular functions, and promotes metabolic rewiring [54]. Indeed, distinct ribosomal protein expression patterns have been described between normal and cancerous tissues where ribosomal patterns are both tissue- and tumour-specific and correlate with clinicopathological features [55]. A recent study showed that alternative splicing of ribosomal proteins is spatially arranged in GBM tissues (i.e., tumour periphery and core), and variations in ribosome protein components create complexes with distinct cellular functions that promote the gene expression programs associated with different GBM phenotypes [56]. Four of the five best performing uEV GBM diagnostic proteins resolved here are ribosomal subunits (RP13A, RPL18, RPL28, and RPS8). Although changes in ribosomal protein subunits were not observed across the GBM clinical cohorts here, their over-representation in GBM uEVs relative to HC is interesting and should be further investigated to determine whether these patterns are specific to GBM, and if uEVs hold the capacity to delineate GBM tumours by their genetic determinants. As numerous ribosomal protein isoforms are implicated in tumorigenesis, metastasis and therapeutic resistance [54], their presence in uEV cargo may instead represent a pan-cancer marker.

### uEV proteins can distinguish GBM patients at different clinical timepoints

Strikingly, uEV protein levels were able to differentiate GBM patients at different clinical timepoints, including patients with primary and recurrent tumours. Of note, GRN (protein name, *progranulin*) and PSAP (*prosaposin*) displayed significant differential abundance between all three clinical timepoints (Pre-OP, Post-OP and REC; Fig. 3b) and thus are putative candidate biomarkers of GBM tumour burden, tumour recurrence and treatment resistance. GRN is a pleiotropic growth factor with important roles in several physiological processes; GRN deficiency is associated with a broad range of pathological conditions affecting the brain, such as frontotemporal dementia. GRN is upregulated in neoplastic tissues, and has a pro-tumorigenic role by promoting cancer cell proliferation, migration, invasiveness, anchorage-independent growth, modulation of the tumour microenvironment, immune evasion and resistance to chemotherapy [57]. GRN is a known glioma-associated growth factor [58], its overexpression in GBM tumours and prognostic significance is well established [59]. Excitingly, a recent study demonstrated that GRN promotes temozolomide resistance of GBM cells via modulating DNA damage repair pathways and inducing cancer stemness [60]. Here, GRN displayed superior individual biomarker performance (AUC = 0.9841) when classifying uEVs from GBM patients with a tumour recurrence following standard treatment with the STUPP protocol (radiotherapy plus concomitant and adjuvant temozolomide). This interesting marker certainly warrants further investigation in larger clinical cohorts including assessments to determine whether GRN can outperform radiological surveillance (i.e., distinguish patients with tumour recurrences from those with pseudoprogression and/or radiation necrosis).

PSAP is a highly conserved glycoprotein that mediates sphingolipid and ceramide metabolism [61], can exert neurotrophic effects, and its overexpression and secretion are associated with tumorigenesis [62]. Studies have reported high PSAP expression in clinical glioma specimens, glioma-stem cells and cell-lines [62, 63] with PSAP expression and secretion highest in mesenchymal tumours, the most aggressive transcriptional subtype of GBM [64]. We previously reported prosaposin as a GBM EV signature protein and showed that prosaposin levels in GBM-EVs have a significant, positive correlation to in vitro GBM cell invasion [40]. Recently, PSAP was shown to promote GBM invasion and epithelial–mesenchymal transition (EMT)-like processes via the TGF-β1/Smad signaling pathway [64], a strategy used by lung cancer cells to acquire radioresistance [65]. GBMs most frequently shift to the mesenchymal phenotype on relapse [66], and the observed high levels of PSAP in uEVs in REC samples here and significant association with treatment resistant GBM recurrence may reflect this. In stark contrast, contradictory findings of significantly lower PSAP levels have been observed in GBM plasma EVs relative to non-tumour controls [5]. In the context of a GBM diagnostic assay, it may be more valuable to assess protein expression patterns as GBM biomarkers than fixed abundance values, as EV-associated biomarker levels vary across different biofluids.

Together with GRN, a logistic regression model also selected ITM2B (*integral membrane 2B*) as the two best performing uEV biomarkers associated with GBM recurrence (AUC = 1.0). Highly expressed in uEV from GBM REC patients here, ITM2B is a transmembrane protein and known tumour suppressor that triggers p53-independent apoptosis [67]. While the exact mechanisms are still unclear, ITM2B was identified as a downstream effector of miR-143 in GBM cells [67], which has a demonstrated role in GBM invasion [68]; the specific inhibition of miR-143 reduced GBM tumour growth and progression in vivo [67]. While further validation studies are mandatory to assess the sensitivity and specificity of ITM2B, GRN and PSAP as uEV biomarkers, these early results highlight the promise of our approach for a GBM liquid gold biopsy.

### Methodological considerations and study limitations

Ultracentrifugation was selected as it is the current gold standard method for isolating EVs from large volumes of starting fluid and recovers high EV-yields [37]. Alternative uEV isolation approaches, such as size exclusion chromatography (SEC), precipitation or density gradient ultracentrifugation, may improve the purity of EV preparations, though EV-yields are generally lower [37]. The ultracentrifugation method allowed total uEV populations to be isolated for proteomic analysis. While it may be advantageous to capture GBM-specific EVs from the circulation using known markers, such as EGFR/EGFRvIII, beyond the direct impacts on cortical tissues, GBM inflicts a substantial systemic toll [69] that is perhaps better capitulated in total circulating EV populations. As such, analysing total EV populations from the circulation or urinary system may offer important indications of immune status and treatment side effects, with potential clinical uses.

While the urinary proteome is less complex than the blood [70], the highly-abundant urine protein uromodulin (Tamm Horsfall Protein; THP) commonly co-isolates with uEVs and can impact the reliability and reproducibility of LC-MS/MS biomarker discovery analyses. To reduce THP contamination and improve EV recovery from the urine, our method included a Tris(2-carboxyethyl)phosphine (TCEP) reduction of THP during EV isolation [13]. Despite this, THP remained the most abundant protein in the uEV isolates sequenced by initial shotgun LC-MS/MS analyses (data not presented); a DIA-MS approach in conjunction with a highly specific data extraction strategy was therefore employed to improve the proteome coverage and facilitate comprehensive uEV proteomic analysis [71]. DIA-MS acquisitions of the GBM uEVs were aligned to a high-quality custom GBM spectral library that contains 8602 proteins derived from brain tumour tissues, cells and EVs, as well as other cancer lesions [5]. Here, a library-based approach was favoured over library-free methods to maximise the specificity and confidence of proteomic identifications for the complex uEV specimens. Library-free methods often rely on in silico digestion of a protein database to generate precursor ions [72], which offers the flexibility to analyse diverse sample types and to identify proteins that are otherwise absent in a spectral library. However, they result in lower identification confidence and have increased computational requirements [73].

While the uEV changes may offer a true reflection of post-operative GBM states, it is possible that urine samples were collected too soon after surgery (average sampling time was 17.8 h) to accurately reflect a successful reduction in ‘tumour burden’ post-operatively. Generally, circulating-EVs have a short half-life (4–24 h, depending on where they disseminate) and can therefore reflect rapid bodily changes [74]. However, in this study, it is possible that the uEVs may not have had sufficient time to reflect a ‘reduced tumour burden’ signature or, may instead reflect signatures that are unrelated to GBM. Post-operative uEV proteomes are likely impacted by surgical stress, a disrupted blood-brain-barrier, general anaesthesia, and/or other related clinical factors [75]. uEV biomarkers are particularly vulnerable to changes in kidney function and hydration status; the perioperative blood biochemistry profiles of the GBM patients studied were stable, suggesting that kidney function and hydration had limited impact on resolved uEV biomarkers. Future post-operative urine samples could be tested at different time-points, e.g., 48 h, 72 h or 1-week after surgery, to determine the optimal sampling times for ‘tumour burden’ biomarker discovery. Further, regular follow-up urine sampling would allow for a temporal assessment of biomarker changes during treatment, and importantly during tumour surveillance for detecting GBM recurrences early. Unfortunately, we were unable to collect catheterised urine samples from healthy controls to compare with GBM patients so cannot discern the impact of catheterisation on uEV proteomes. Future studies will assess the impact of the different urine collection methods on uEV biomarker levels, as sampling from GBM patients on tumour surveillance will practicably be midstream specimens.

### Future directions and concluding remarks

This promising feasibility study highlights the potential for a uEV-based liquid biopsy for GBM. Using a DIA-LC-MS/MS approach, a comprehensive and in-depth proteomic characterisation of uEVs was performed, substantiating urine as a viable source of EV-derived biomarker proteins. uEV proteomic signatures specific to GBM patients were determined and differentially abundant uEV proteins corresponding to tumour burden and recurrent progression were identified. Putative uEV proteomic biomarkers corresponding to different GBM clinical timepoints included previously defined GBM-EV proteomic biomarkers from cell culture, neurosurgical fluids, and blood-plasma. Previous reports of EV-derived miRNA biomarkers in other biofluids [1, 4] provide a sound rationale for further exploration of uEV miRNA biomarkers, particularly given that EV-derived miRNAs have been shown to augment GBM progression, and influence and contribute to chemo- and radio-resistance [76]. Our future investigations will include larger, longitudinal cohorts of urine samples captured from patients at multiple clinical time-points, including at treatment baseline, end-of-treatment, and either pathology-proven tumour recurrence or pseudoprogression. With accompanying clinicopathologic and radiological information, validation studies will test the clinical utility of the putative liquid gold biomarkers resolved here and provide greater insight into uEV biomarker dynamics.

### Supplementary information

Supplementary Table 1

Supplementary Tables 2–5

Supplementary Figure 1

Supplementary Figure 2

Supplementary Figure 3

Supplementary Figure 4

Supplementary Figure 5

## Data Availability

The SWATH spectral library is available in PeptideAtlas with the identifier PASS01487. Normalised data used for statistical analysis is provided in Supplementary Table 4. The mass spectrometry proteomics data have been deposited to the ProteomeXchange Consortium via the PRIDE [34] partner repository with the dataset identifier PXD046511.
