# Supplementary material for: Glioblastoma biomarkers in urinary extracellular vesicles reveal the potential for a ‘liquid gold’ biopsy
Source: Br J Cancer. 2024 Jan 11;130(5):836–51. doi: 10.1038/s41416-023-02548-9 (PMC10912426; doi:10.1038/s41416-023-02548-9)
Supplement: Supplementary file 1 — Supplementary Table 1 [file 41416_2023_2548_MOESM1_ESM.docx]

Supplementary Table 1: Participant demographics and sample information

| Experimental cohorts | Urine ID | Age | Gender | Occurrence | Extent of Resection | Urine starting vol. (mL) |
| --- | --- | --- | --- | --- | --- | --- |
| Pre-OP  Primary GBM  (n=17);  ** denotes samples*  *with matched*  *Post-OP* | UG26 * | 46 | Male | De novo (primary) |  | 35 |
|  | UG30 * | 66 | Female | De novo (primary) |  | 50 |
|  | UG48 * | 72 | Male | De novo (primary) |  | 35 |
|  | UG52 * | 56 | Male | De novo (primary) |  | 35 |
|  | UG62 * | 43 | Male | De novo (primary) |  | 30 |
|  | UG70 * | 76 | Female | De novo (primary) |  | 30 |
|  | UG28 * | 60 | Male | De novo (primary) |  | 30 |
|  | UG42 * | 49 | Male | De novo (primary) |  | 35 |
|  | UG91 * | 42 | Male | De novo (primary) |  | 60 |
|  | UG18 | 73 | Female | De novo (primary) |  | 30 |
|  | UG56 | 57 | Female | De novo (primary) |  | 30 |
|  | UG72 | 74 | Female | De novo (primary) |  | 30 |
|  | UG46 | 60 | Female | De novo (primary) |  | 30 |
|  | UG88 | 56 | Male | De novo (primary) |  | 30 |
|  | UG24 | 92 | Female | De novo (primary) |  | 60 |
|  | UG22 | 62 | Male | De novo (primary) |  | 30 |
|  | UG76 | 68 | Male | De novo (primary) |  | 25 |
| Post-OP  Primary GBM (matched, n=9) | UG27 | 46 | Male | De novo (primary) | Gross total | 60 |
|  | UG31 | 66 | Female | De novo (primary) | Gross total | 50 |
|  | UG49 | 72 | Male | De novo (primary) | Gross total | 35 |
|  | UG53 | 56 | Male | De novo (primary) | Gross total | 35 |
|  | UG63 | 43 | Male | De novo (primary) | Gross total | 30 |
|  | UG71 | 76 | Female | De novo (primary) | Gross total | 30 |
|  | UG29 | 60 | Male | De novo (primary) | Gross total | 30 |
|  | UG82 | 49 | Male | De novo (primary) | Gross total | 30 |
|  | UG92 | 42 | Male | De novo (primary) | Gross total | 60 |
| Pre-OP  GBM Recurrence (n=7) | UG20 | 77 | Female | Relapse(secondary) |  | 60 |
|  | UG60 | 27 | Female | Relapse (secondary) |  | 60 |
|  | UG79 | 45 | Male | Relapse (secondary) |  | 30 |
|  | UG93 | 71 | Female | Relapse (secondary) |  | 30 |
|  | UG73 | 47 | Male | Relapse (secondary) |  | 20 |
|  | UG83 | 69 | Male | Relapse (secondary) |  | 50 |
|  | UG87 | 71 | Female | Relapse (secondary) |  | 50 |
| Healthy,  non-cancer CONTROL  (n=14) | UH8 | 66 | Male |  |  | 50 |
|  | UH9 | 63 | Female |  |  | 100 |
|  | UH10 | 73 | Male |  |  | 100 |
|  | UH11 | 68 | Female |  |  | 100 |
|  | UH14 | 75 | Male |  |  | 100 |
|  | UH16 | 72 | Male |  |  | 100 |
|  | UH17 | 65 | Female |  |  | 100 |
|  | UH18 | 65 | Male |  |  | 100 |
|  | UH19 | 75 | Male |  |  | 100 |
|  | UH20 | 69 | Female |  |  | 100 |
|  | UH21 | 71 | Female |  |  | 100 |
|  | UH22 | 71 | Male |  |  | 100 |
|  | UH23 | 70 | Female |  |  | 100 |
|  | UH24 | 69 | Female |  |  | 100 |
