# Supplementary figures and images for: Glioblastoma biomarkers in urinary extracellular vesicles reveal the potential for a ‘liquid gold’ biopsy
Source: Br J Cancer. 2024 Jan 11;130(5):836–51. doi: 10.1038/s41416-023-02548-9 (PMC10912426; doi:10.1038/s41416-023-02548-9)

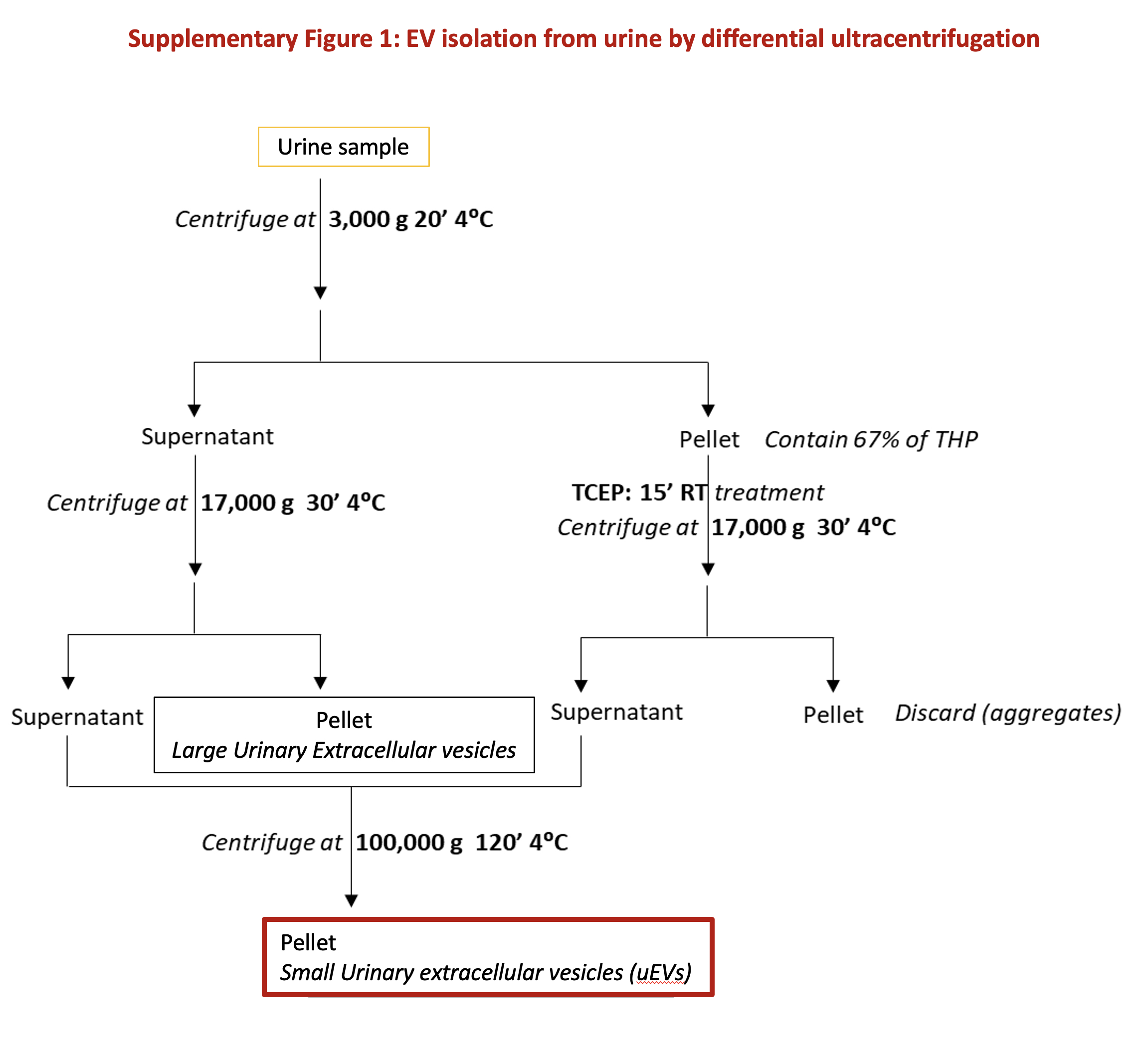

Supplement: Supplementary file 3 — Supplementary Figure 1 [file 41416_2023_2548_MOESM3_ESM.jpg]

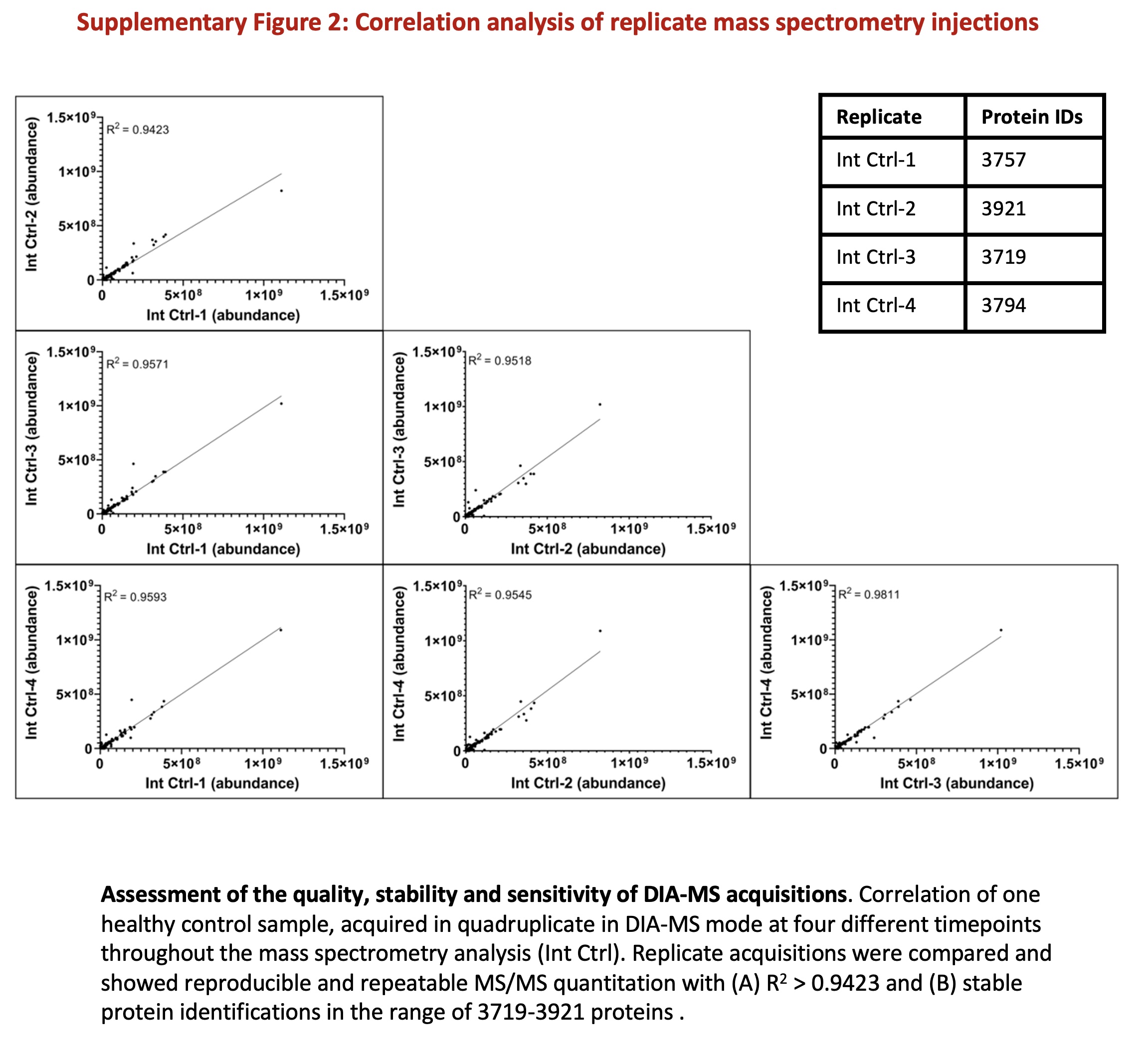

Supplement: Supplementary file 4 — Supplementary Figure 2 [file 41416_2023_2548_MOESM4_ESM.jpg]

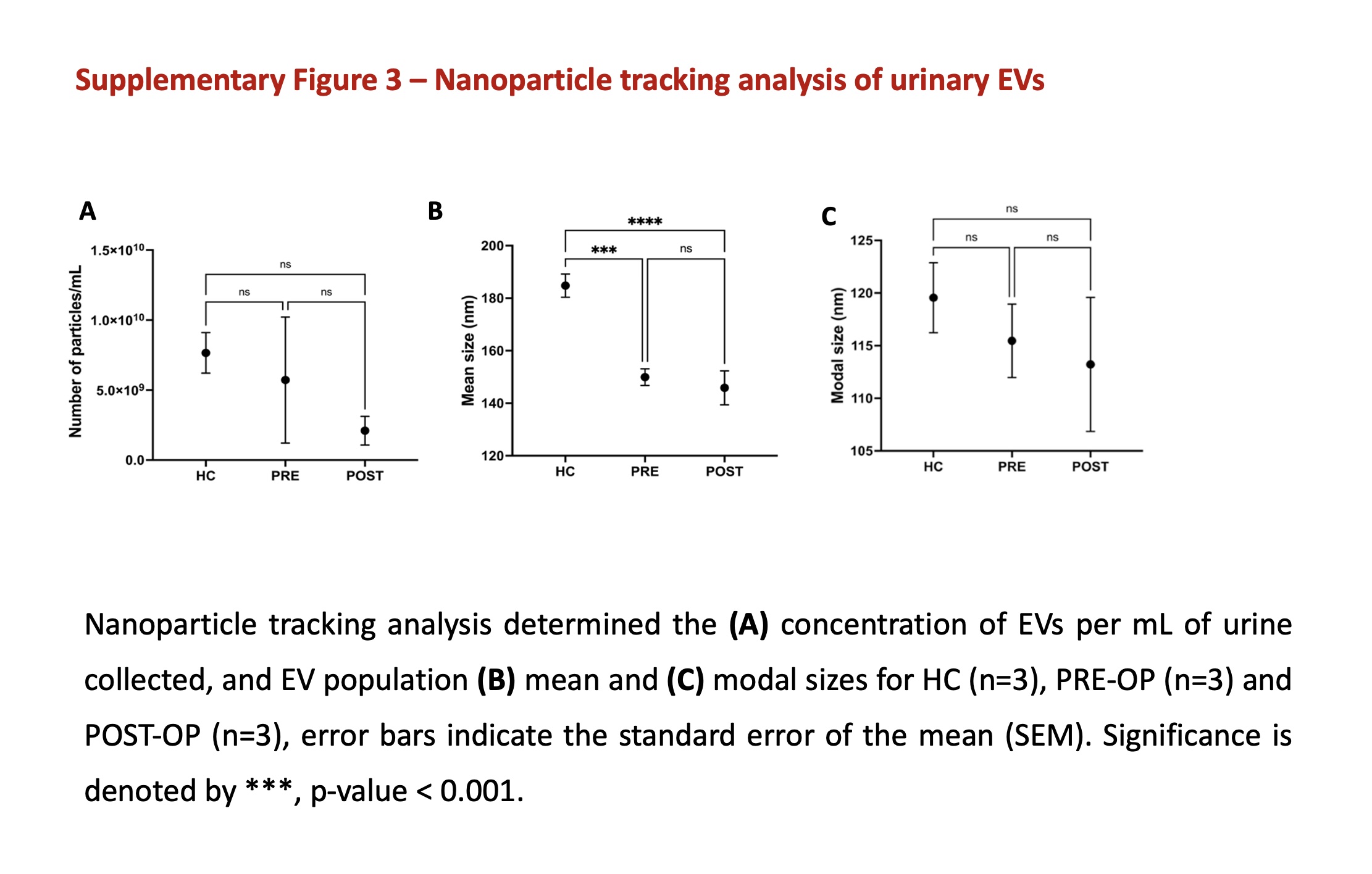

Supplement: Supplementary file 5 — Supplementary Figure 3 [file 41416_2023_2548_MOESM5_ESM.jpg]

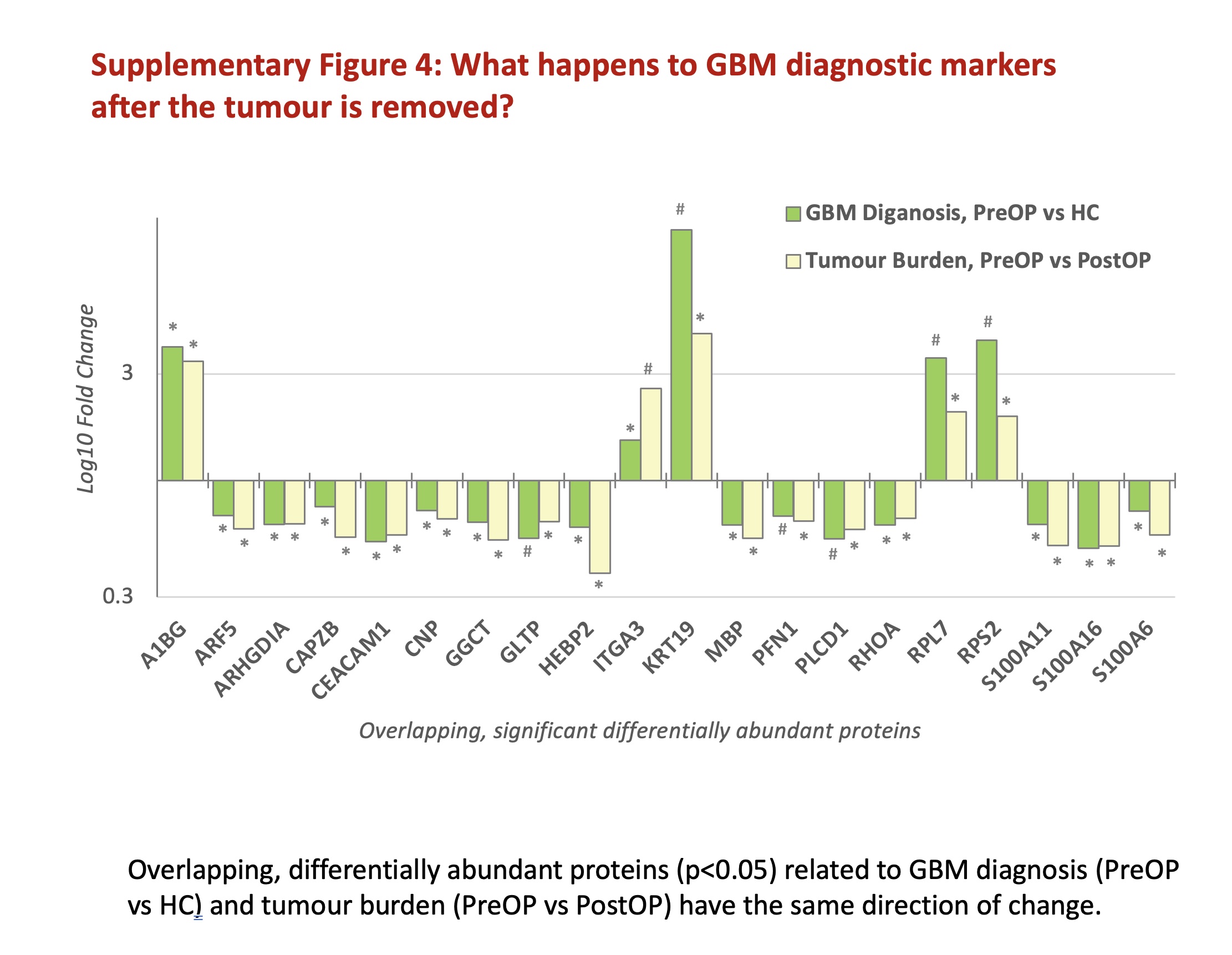

Supplement: Supplementary file 6 — Supplementary Figure 4 [file 41416_2023_2548_MOESM6_ESM.jpg]

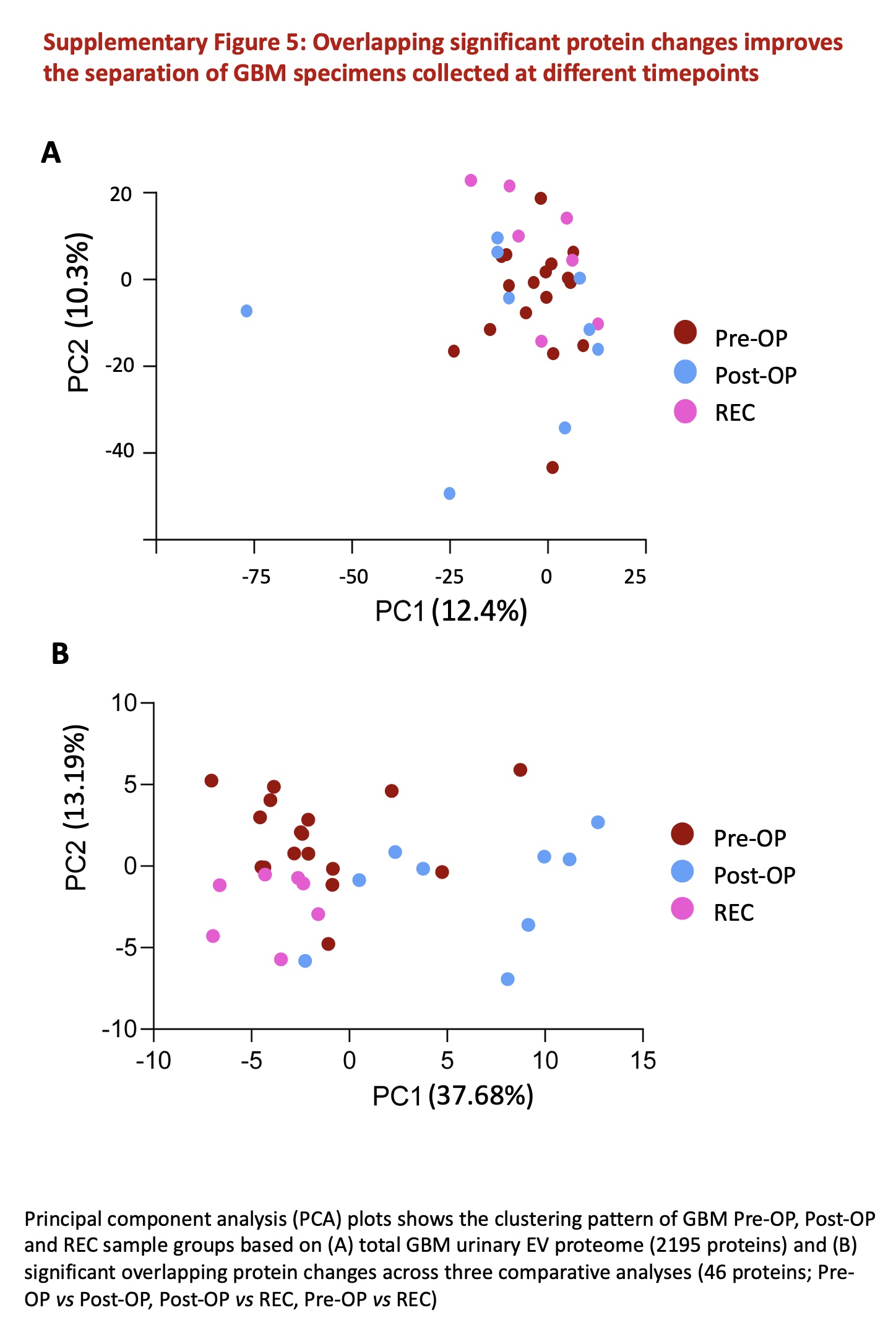

Supplement: Supplementary file 7 — Supplementary Figure 5 [file 41416_2023_2548_MOESM7_ESM.jpg]
